# Long non‐coding RNAE330013P06 promotes progression of breast cancer with type 2 diabetes

**DOI:** 10.1002/jcla.23172

**Published:** 2020-01-06

**Authors:** Runqi Chen, Pengcheng Shi, Yan Zhang, Haiming Wu, Xiaoping Li, Wengfu Yang, Fei Luo, JinmingXue, Liang Yao, Jun Yang, Wangfu Wang, Bo Zhang, Peng Li, Yongmin Miao, Qianjun Wang, Fuguo Tian

**Affiliations:** ^1^ Department of Breast Oncology Shanxi Cancer Hospital Taiyuan China

**Keywords:** breast cancer, cell growth, diabetes, long non‐coding RNAE330013P06

## Abstract

**Background:**

In previous research, we found diabetes rather than obesity was an independent risk factor of breast cancer. However, why diabetes could lead to increased risk of breast cancer patients remains elusive. Long non‐coding RNAE330013P06 has been shown to be upregulated in diabetes, and long non‐coding RNAs generally promote progression of cancer.

**Methods:**

About 200 specimens of breast patients were obtained in previous clinical trial; 34 samples diagnosed as type 2 diabetes in breast cancer patient were enrolled in this research. Blood samples from 36 patients diagnosed as breast cancer without diabetes; 35 diabetic patients and 35 healthy peoples were obtained as control. All blood samples were measured by quantitative real‐time PCR (qRT‐PCR). Invasion and migration were tested by Transwell assay. Cell proliferation assay was tested by CCK‐8. Protein analysis was determined by Western blot.

**Results:**

Compared with breast cancer patients without diabetes, diabetic patients without breast cancer and healthy peoples, LncRNAE330013P06 was upregulated in breast cancer patient with diabetes. Furthermore, of 34 breast patients, high LncRNAE330013P06 expression was significantly associated with family history, tumor‐node‐metastasis stage and lymph node metastasis. E33 promoted cancer cell growth in vitro via downregulation of P53.

**Conclusion:**

Upregulation of LncRNAE330013P06 driven by type 2 diabetes is one of the factors which promoted progression of breast cancer.

## 1 INTRODUCTION

Breast cancer remains to be a major cause of cancer death in females worldwide.^1^ Like several epithelial cancers, breast cancer is also a heterogeneous disease characterized by multiple pathological subtypes.^2^

Traditional views thought diabetes especially type 2 diabetes was associated with increased risk of breast cancer, because of higher body mass index (BMI). Our previous research showed that diabetes is an independent factor for risk of breast cancer rather than BMI.^3^ Nevertheless, there were few breast patients with diabetes 1 that might be accounted for shorter life span of patients or its lower BMI. It has been demonstrated diabetic patients with breast cancer receiving metformin and neoadjuvant chemotherapy have a higher pCR rate than do diabetics not receiving metformin.^4^ So, the underlying mechanism remains unknown. Upregulation of IGF‐1 of plasm is an explanation why diabetes 2 would increase the risk of breast cancer.^5^ But, some other researches seemed not to support this explanation.

Recently, long non‐coding RNAs (LncRNA) of plasma, that were thought to promote cancer development, were found to take a significant role in diabetes 2 progression.^6^ Long non‐coding RNAs are non‐coding RNAs that are longer than 200 nucleotides, and many of them were demonstrated to play a pivotal role in the development and progression of many diseases including cancer.^7^, ^8^ A series of LncRNAs have been demonstrated as oncogenes and/or tumor suppressor genes.^9^ Furthermore, a number of recent studies suggested the involvement of LncRNAs in cancer development, including in breast cancer.^10^, ^11^ BC200 LncRNA plays an oncogenic role in estrogen‐dependent breast cancer by targeting for attenuating deregulated cell proliferation and possibly serves as a prognostic marker.^12^ Some of the candidate LncRNAs that are shown to be deregulated in breast cancer have the potential to be used as prognostic biomarkers.^13^

For breast patients with diabetes 2, it is reported there are crosslink molecular factors in serum of patient plasma between these two diseases. So to find whether there is any kind of LncRNA in these two diseases and what role it takes in disease progression became very important to understand molecular mechanism of these two diseases. H19 (H19 Imprinted Maternally Expressed Transcript) is an RNA Gene, and is affiliated with the non‐coding RNA class. Diseases associated with H19 include Wilms tumor 2 and Beckwith‐Wiedemann syndrome.^14^ MT‐LIPCAR (Mitochondrially Encoded Long Non‐Coding Cardiac Associated RNA) is an RNA gene and is affiliated with the non‐coding RNA class. Diseases associated with MT‐LIPCAR include congestive heart failure and myocardial infarction.^15^ GAS5 (growth arrest specific 5) is an RNA gene and is affiliated with the non‐coding RNA class. Diseases associated with GAS5 include inflammatory bowel disease and autoimmune disease.^16^ LncRNAs, E330013P06 (hereafter referred to as E33) showed that it is significantly upregulated in vivo and in vitro in macrophages under T2D conditions.^17^ All these four types of non‐coding RNA could be tested to upregulate in serum of some diabetic patients. So, we tested all these four types of RNA in serum of breast cancer patients with and without type 2 diabetes, and we found E33 is higher in breast cancer patients with type 2 diabetes than in those without it. Further, E33 is higher in breast cancer patients with type 2 diabetes than those healthy ones or those patients only with type 2 diabetes without breast cancer. In vitro, T47D and MDA‐MB‐231 showed E33 transfection increased cell proliferation rather than migration and invasion. E33 downregulated P53, but not other protein factors in downstream pathways. All findings above showed E33 is a long non‐coding RNA driven by diabetes that promotes development of breast cancer.

## 2 MATERIALS AND METHODS

### 2.1 Breast cancer blood sample and cell culture

Plasma samples were prepared using the blood extracted from total 105 patients and 35 healthy ones. The study was approved by ethics committee of medical faculty of the Shanxi Cancer Hospital and the First Affiliated Hospital of Shanxi Medical University, and written informed consent was taken from all included patients or their families in accordance with the Declaration of Helsinki. About 34 breast cancer patients with type 2 diabetes and another 36 breast patients without diabetes were enrolled in this research, and we collected fresh plasma samples from September 2012 to November 2013 at Shanxi Cancer Hospital. And 35 paired diabetic patients without breast cancer were enrolled as control in this research, and we collected fresh plasma samples from at the First Hospital of Shanxi Medical University. About 35 healthy people plasma samples were obtained from Medical Examination Center of Shanxi Cancer Hospital. Fresh peripheral blood samples (3 mL) were collected in EDTA tubes and then centrifuged (3000 × g for 10 minutes). Subsequently, the plasma samples were stored at −80°C.

Clinical characteristics were collected, which was individuals including age, family history, tumor differentiation, tumor‐node‐metastasis stage (TNM), lymph node status, estrogen receptor (ER), progesterone receptor (PR), and human epidermal growth factor receptor‐2 (Her2). Tumors were staged according to the tumor‐node‐metastasis (TNM) staging system of the International Union Against Cancer. There was no radiotherapy, chemotherapy, or targeted therapy prior to the operation.

### 2.2 cell culture

Two breast cancer cell lines (MDA‐MB‐231, T47D) purchased from Chinese Academy of Sciences. All cells were cultured in DMEM medium (RSBM) containing 10% of fetal bovine serum (FBS) (RSBM) at 37°C with 5% CO_2_.

### 2.3 Cell transfection

MDA‐MB‐231 and T47D cells were transplanted into 24‐well plates incubated 12 hours in advance, so that the confluence of the cells reached about 30%, and 1 mL of the whole culture medium was added. E330013P06 cDNA was cloned in the lentiviral expression vector pEZ‐LV105 (Kindly provided by Qihong, Fudan University) and verified by DNA sequencing. Plasmid pEZ‐LV105 expressing EGFP (LVGFP) was used as vector control. HEK293T cells were co‐transfected with lentiviral vector plasmids and three packaging plasmids. Cell culture supernatants containing lentiviruses were collected 48‐ to 96‐h post‐transfection and concentrated using Lenti‐Kit (Takara). MDA‐MB‐231 and T47D were transduced with the indicated lentiviral vectors in the presence of Polybrene (8 μg/mL), and cells resistant to puromycin (2 μg/mL) were selected for further analysis.

### 2.4 Reverse transcription quantitative real‐time PCR (qRT‐PCR)

Total RNAs of plasma were extracted by TRIzol reagent (Invitrogen) according to the manufacturer's instructions and stored immediately at −80°C. Then, total RNA was quantified using a Nanovue (GE). Reverse transcription (RT) reaction was performed in the Bio‐Rad C1000 PCR System (Bio‐Rad). The E33 was determined using SYBR Mix (Tiangen) via Step‐One System (Applied Biosystem). The processes of qRT‐PCR reaction were conducted in a volume of 20 μL mixed with 30 ng cDNA templates and gene‐specific primers for E33^18^ (forward primer 5′‐TCTTTCTCACAGGCCGCATT‐3′, reverse primer 5′‐TGATTTCTCCACGGTCAGGC‐3′), LncRNA H19^19^ (forward primer TGCTGCACTTTACAACCACTG, reverse primer ATGGTGTCTTTGATGTTGGGC), LncRNA LIPCAR^14^ (forward primer TAAAGGATGCGTAGGGATGG, reverse primer TTCATGATCACGCCCTCATA) and LncRNA GAS5^20^ (forward primer TTTCGAGGTAGGAGTCGACTCCTGTG, reverse primer TTTTTTTTTTTTTTTTTTTGTATTGCAAA) and internal control GAPDH^21^ (forward primer 5′‐CTCTGCTCCTCCTGTTCGAC‐3′, reverse primer 5′‐GCGCCCAATACGACCAAATC‐3′) for each well. The data were analyzed using 2^−ΔΔCt^ method. The experiments were conducted in triplicate, and the mean value was obtained.

### 2.5 Cell proliferation assay

The cells were cultured in 96‐well plates. 10 μL CCK‐8 solution (Dojindo) was added to each well and incubated for 2 hours at 37°C. The absorbance was measured at 450 nm on a microplate reader according to the manufacturer's instructions. Three multiple holes were set in each hole to calculate the proliferation rate of the two groups of cells.

### 2.6 Cell cycle assay

The cells were cultured in 24‐well plates for 48 hours. The cells were collected and fixed with cold 70% ethanol at −20℃ overnight. Then, the cells were washed and resuspended in cold PBS and incubated at 37°C for 30 minutes with 10 mg/mL RNase and 1 mg/mL propidium iodide (Beyotime). RNA content was detected by flow cytometry (BD). The percentage of cells at G_0_/G_1_ and S phases was analyzed using the Cell Quest acquisition software (BD Bioscience).

### 2.7 Transwell invasion assay

The invasion assay was performed in Transwell (Corning Incorporated) plates. The upper wells were coated with Matrigel (BD Biosciences) at 37°C for 24 hours in a 5% CO_2_ incubator. The cells were starved without serum for 24 hours, and then, 500 μL of cell suspension containing 5 × 10^5^ cells were seeded in the upper chamber. Culture medium supplemented with 10% FBS (750 µL) was added into the lower chamber and incubated for 48 hours. After the incubation, the cells on the upper surface were wiped with a cotton swab. The migrated cells on the lower surface were washed with PBS, fixed in with cold methanol, and stained with crystal violet. The cells that invaded to the lower surface were counted in every five high power fields (100×) under a microscope.

### 2.8 Cell apoptosis assay

Apoptosis was quantitated by flow cytometry after staining cells with FITC‐labeled Annexin‐V (AV) (Beyotime) and propidium iodide (PI). Cells were harvested and centrifuged at 1000*g* for 5 minutes; the cells were washed twice with PBS and then resuspend in 100 µL of Annexin‐V binding buffer. About 1 µL Annexin‐V and 5 µL PI were added to the samples and incubated in the dark for 15 minutes. Samples were kept on ice after incubation until FACS analysis was performed. Results were expressed as mean ± S.D (n = 3), and *P* < .05 was considered to be statistically significant.

### 2.9 Western blot assay

Protein Mycodin, KLF4, ELK‐1 and P53^22^, ^23^, ^24^, ^25^ were separated by 10% SDS‐PAGE and transferred into polyvinylidene fluoride membranes (Millipore). After blocking with 5% non‐fat milk overnight at 4°C, the membranes were incubated with rat anti‐Mycodin, anti‐KLF4, anti‐ELK‐1, and anti‐P53. Then, membranes were washed and incubated with HRP‐conjugated rabbit anti‐rat IgG as the secondary antibody. Protein bands were detected using enhanced chemiluminescence kit (ECL, Pierce).

### 2.10 Statistical analysis

We used Statistical Product and Service Solution (SPSS) software 18.0 (SPSS Inc) to analyze the experimental data. A paired *t* test was employed to evaluate LncRNAE330013P06 expression in plasma samples. By one‐way analysis of variance (ANOVA), we further evaluated the correlation between E33 levels and clinicopathologic factors of patients with diabetes. Five‐year overall survival rates for breast cancer patients with diabetes and the risk of breast cancer in diabetes also be assessed (Table 1).

**Table 1:** Patients of breast cancer with diabetes (B&D), breast cancer without diabetes (B without D), diabetes (D), and healthy ones (H)

Factor	B&D	B without D	D	H
Age (y)	24 (70.6)	24 (66.7)	24 (68.6)	24 (68.6)
<65	10 (29.4)	12 (33.3)	11 (31.4)	11 (31.4)
≥65				
Tumor grade				
Ⅰ‐Ⅱ	23 (66.7)	26 (72.2)		
Ⅲ	11 (32.4)	10 (27.8)		
TNM stage				
Ⅰ	9 (26.5)	9 (25.0)		
Ⅱ	14 (41.2)	14 (38.9)		
Ⅲ	8 (23.5)	8 (22.2)		
Ⅳ	3 (8.8)	5 (13.9)		
ER status				
+++	10 (29.4)	11 (30.6)		
++	10 (29.4)	10 (27.8)		
+	7 (20.6)	8 (22.2)		
−	7 (20.6)	7 (19.4)		
PR status				
+++	11 (32.4)	10 (27.8)		
++	10 (29.4)	10 (27.8)		
+	6 (17.6)	9 (25.0)		
−	7 (20.6)	7 (19.4)		
Her‐2 status				
+++	8 (23.5)	8 (22.2)		
++	6 (17.6)	6 (16.7)		
+	6 (17.6)	8 (22.2)		
−	14 (41.2)	14 (38.9)		
BMI	24 ± 1.5	26.3 ± 2.1	26.2 ± 3.1	23.1 ± 1.2
Insulin		16 (44.4)	16 (45.7)	

## 3 RESULTS

### 3.1 Upregulation of LncRNAE330013P06 in breast cancer specimens with diabetics

**Figure 1:**
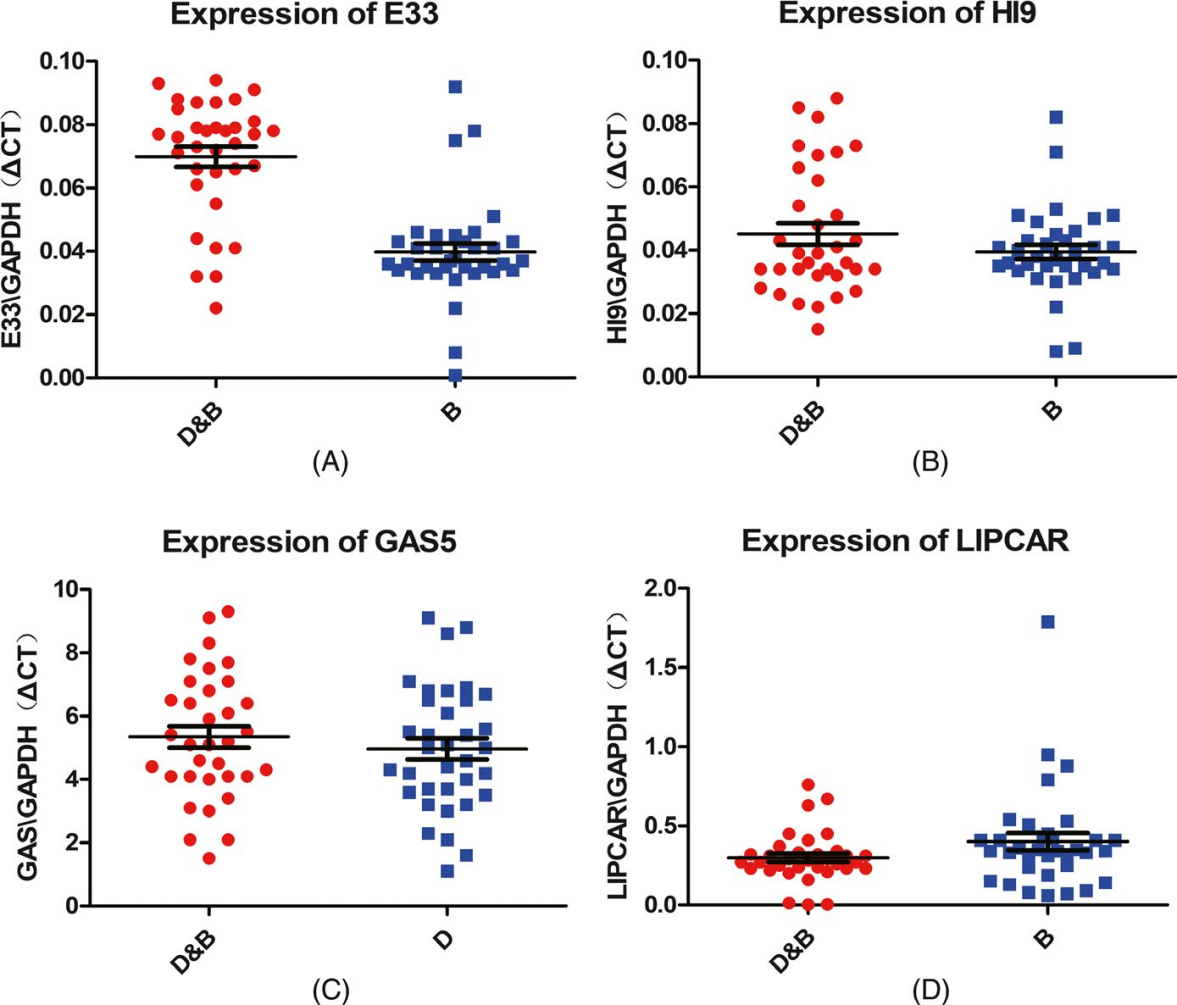
(A‐D) Relative levels of four LncRNA/GAPDH in plasma samples of breast cancer patients with diabetes (D&B) and breast cancer patients without diabetes (B). A paired *t* test was performed, indicate *P* < .001. D&B was breast cancer patients with diabetes and B was breast patients without diabetes

To confirm the higher E33 expression in breast cancer induced by diabetes, we selected four LncRNAs (H19, E33, LIPCAR, and GAS5) that have been shown to be associated with diabetes. These four LncRNAs expression was determined by qRT‐PCR in breast cancer patients with diabetes and breast cancer patients without diabetes. All RNAs were extracted from serum of blood samples. And cDNA was obtained by RT‐PCR, and then RNA was tested by real‐time PCR. Results were averaged. It was very clear that E33 was the only one overexpressed in breast cancer patients with diabetes far more than those without diabetes in four types of LncRNA, about 78% (Figure 1A).

**Figure 2:**
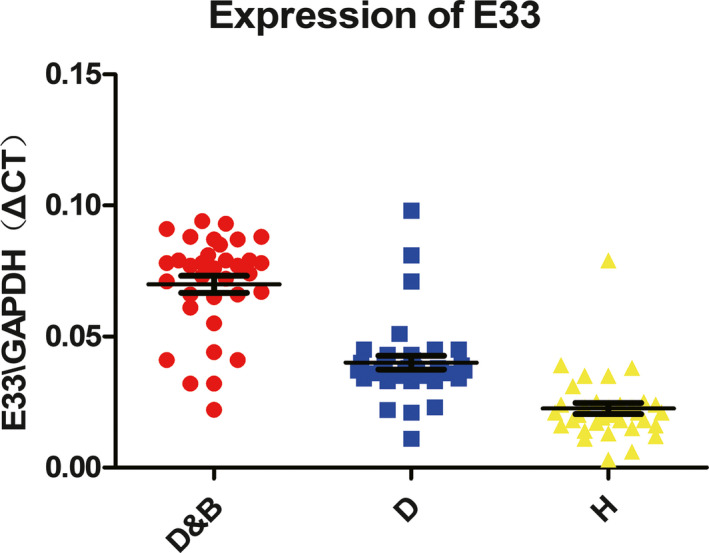
(A and B). Relative levels of E33/GAPDH in plasma samples of breast cancer patients with diabetes (D&B), diabetic patients without breast cancer (D) and healthy people (H). A paired *t* test was performed, indicate *P* < .001

To investigate whether higher expression of E33 in plasma in breast cancer patients with diabetes existed in diabetic patients without breast cancer, we used a qRT‐PCR to detect the expression levels of E33 of 35 blood samples from patients who diagnosed as type 2 diabetes without breast cancer and 35 healthy people in Medical Examination Center (MEC). The results showed that E33 was highly expressed just in breast cancer patients with diabetes rather than other groups (*P* < .001, Figure 2) (Table 2).

**Table 2:** Expression of E33 of breast cancer patients with diabetes

Factor	D&B	E330013P06 expression
Family history		
Absent	28 (82.4)	0.074
Present	6 (17.6)	0.093
TNM stage		
Ⅰ	9 (26.5)	0.068
Ⅱ	14 (41.2)	0.075
Ⅲ	8 (23.5)	0.092
Ⅳ	3 (8.8)	0.101
Lymph node metastasis		
0	11 (32.4)	0.082
1‐3	13 (38.2)	0.067
4‐9	8 (23.5)	0.094
10	2 (5.9)	0.166

### 3.2 LncRNAE330013P06 high‐expression was associated with poor prognosis of breast patients with diabetes

**Figure 3:**
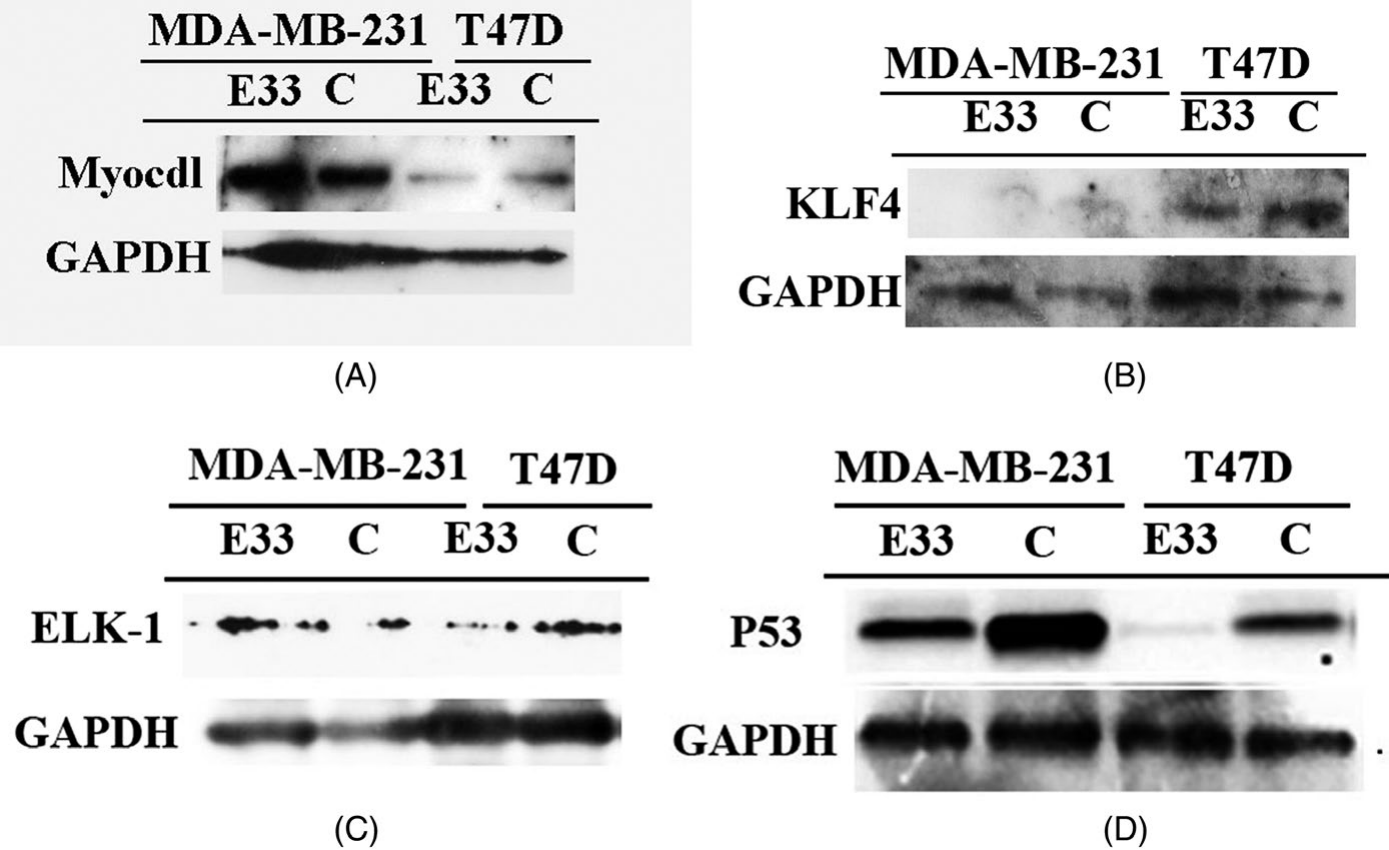
Five years of overall survival (OS) in different stage, Kaplan‐Meier survival curve

High E33 expression was remarkably correlated with TNM stage (*P* = .002), lymph node metastasis (*P* = .015), and family history (*P* = .013), but not correlated with patient's age and tumor grade, as well as ER, PR, and Her2 status (*P* > .05). Furthermore, Kaplan‐Meier survival analysis revealed that high E33 expression was associated with low overall survival (Figure 3). In 34 breast patients with diabetes, the median follow‐up time was 51 months. About 15% cases with high E33 expression got worse in breast cancer.

### 3.3 Proliferation rate of breast cancer cells slows down after gene silencing

**Figure 4:**
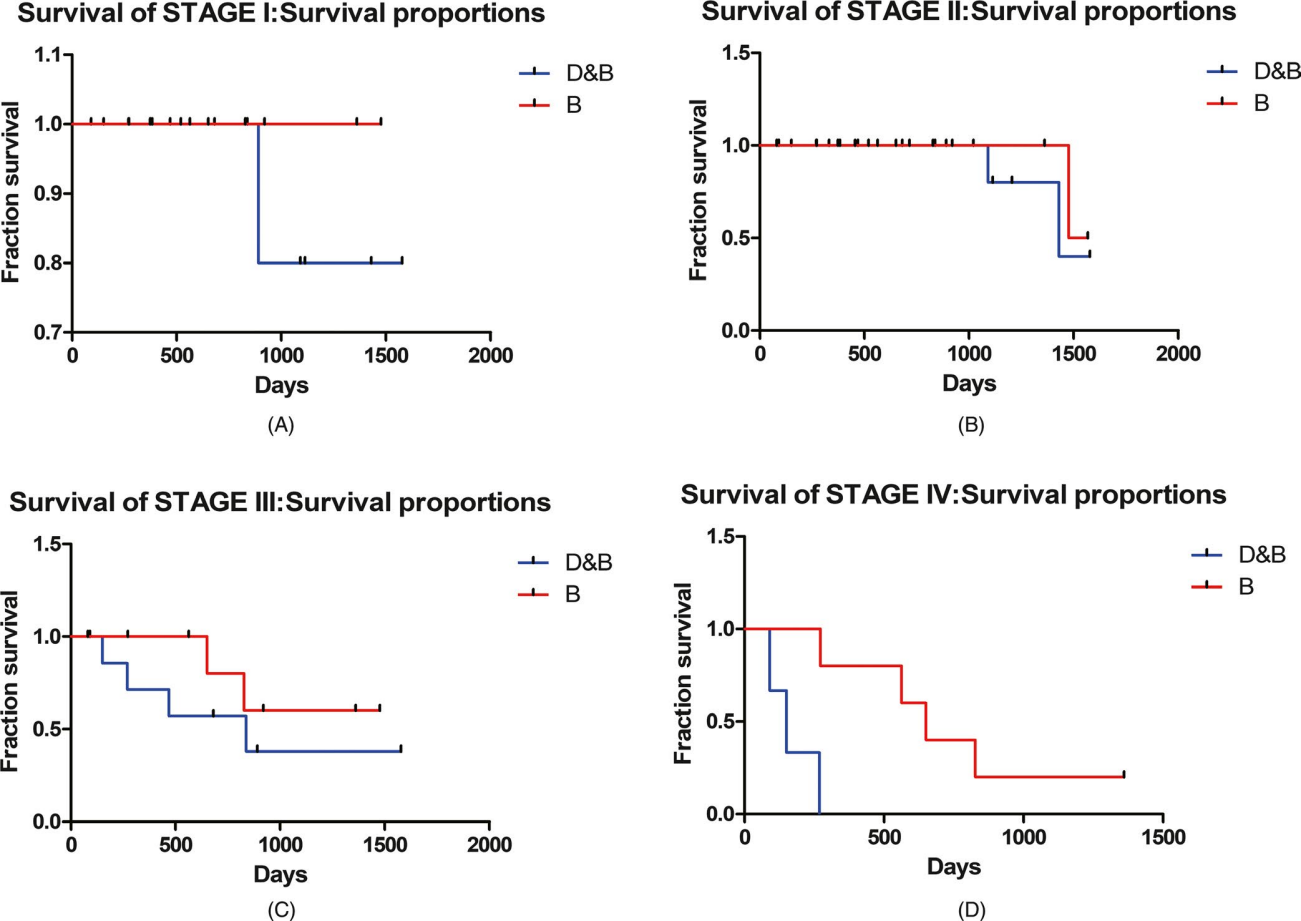
Proliferation (A) and invasion (B) of transfected and control breast cancer cells MDA‐MB‐231 and T47D. E33 transfection in MDA‐MB‐231 and T47D showed by EGFP in inverted fluorescent microscope (C)

After transfection of E33, proliferation of the MDA‐MB‐231 and T47D cell cycle was measured; it was higher than that of the control. The proliferation rate of the E33 overexpression MDA‐MB‐231 cells was also higher than that of the T47D cells (Figure 4A).

These results showed E33 stimulated growth of breast cancer cells. Often, LncRNAs stimulated invasion or inhibited cell apoptosis according to past researches. But, direct transfection of E33 to breast cancer cell lines could promote cell growth.

### 3.4 Invasion number of breast cancer cells, cell cycle, and apoptosis of breast cancer cells associated with E33

At same time, the invasion number of the E33 overexpression MDA‐MB‐231 and T47D cells were almost same with that control, and the invasion rate did not change too much after transfection (Figure 4B).

**Figure 5:**
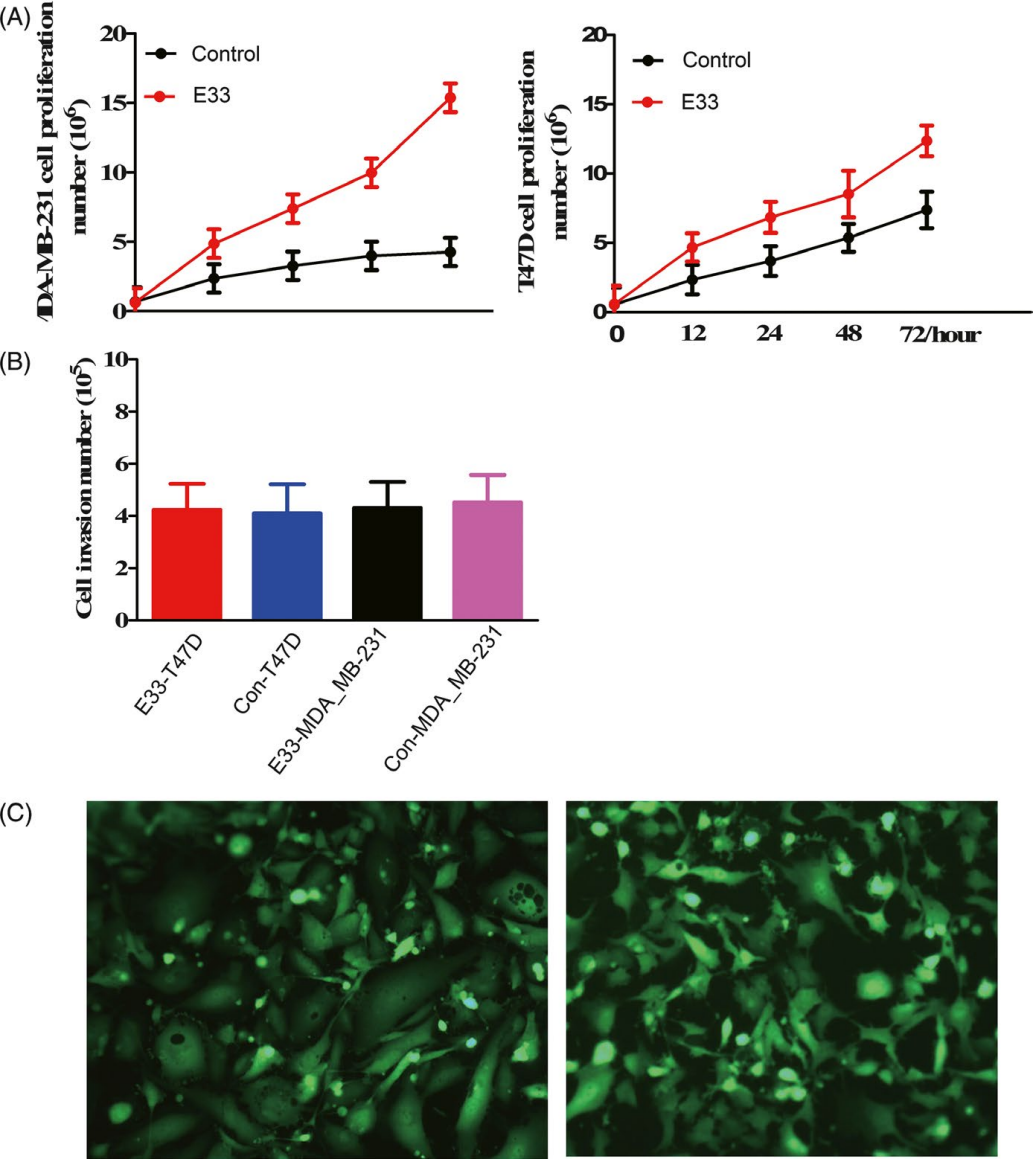
Cell cycle (A) and apoptosis (B) in transfected and control MDA‐MB‐231 and T47D cells

We tested cell cycles in MDA‐MB‐231 and T47D cells transfected and control cells with vector. Transfected cells led to a little higher increase in the fraction in the S phase but not very meaningful. Flow cytometry analysis showed cell apoptosis number of transfected MDA‐MB‐231 and T47D cells showed no difference with control (Figure 5B). These results revealed LncRNA E33 influenced on proliferation rather than cell apoptosis and invasion of breast cancer cells.

### 3.5 E33 promote cell growth via P53

**Figure 6:**
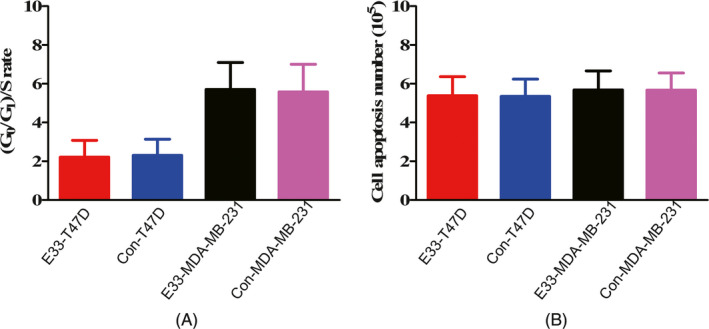
E33 transfection decreases the expression of p53 in MDA‐MB‐231 and T47D cell lines (D). And E33 did not influence expression of Myocdl, KLF4, and ELK‐1(A,B and C)

E33 is miR143/145 like long non‐coding RNA. According to past studies, expression of Myocdl, KLF4, ELK‐1, and P53 might be downstream pathways of E33 (Figure 6). So, we tested the expression of these four proteins. It is very clear with E33 transfection, only P53 was downregulated in these two cell lines (Figure 6D). To confirm these cells transfection, we take photos in inverted fluorescent microscope to ascertain its transfection efficiency (Figure 4C).

The results of Western blot showed that the expression levels of P53 could be downregulated by E33. P53 pathway is the major cause of cell growth by E33.

## 4 DISCUSSION

With development of breast cancer research, new drugs and treatments increase overall survival (OS) of patients in past few decades. Yet, statistics showed very large amount of patients would have a lineal recurrence relation with survival period, even after 10 years, especially for those ER positive patients.^26^ New research finds that the diabetes drug metformin changes stem cancer cells in a way that makes them easier to target with a new form of treatment.^27^ Metformin showed evidenced efficacy for ER positive breast cancer after 10 years. Traditional explanation is metformin decrease BMI and estrogen from body adipose tissue, hence. But new findings render metformin could help treat triple‐negative breast cancer, which is particularly aggressive.^28^ Furthermore, fast mimic diet (FMD) is effective for stage IV patients of breast cancer and type 2 diabetes as well.^29^ All these recent findings showed how significant of crosslinks between diabetes and breast cancer.

Past researches showed that there is a crosslink between type 2 diabetes and breast cancer.^18^, ^30^, ^31^ But often, types of protein factors in serum were studied in past years. IGF‐1 was thought to be the most important crosslink between breast cancer and type 2 diabetes.^32^ But, other researches showed IGF‐1 cannot account for crosslink between breast cancer and type 2 diabetes overall. Are there other factors but not protein more crucial for cancer progression in serum for patients? Our research showed non‐coding RNA takes this role.

LncRNAE330013P06 could contribute to cell proliferation and tumorigenesis in diabetes.^17^ However, clinical relevance of E33 in breast cancer with type 2 diabetes remains unknown. In the present study, our results indicated that LncRNAE330013P06 expression was increased in breast patients with type 2 diabetes, plasma compared with corresponding normal plasma, plasma of breast cancer patients without diabetes, and plasma of patients with type 2 diabetes. These data combined with a previous study showed that E33 was overexpressed in breast patient plasma and promoted cell proliferation of breast cancer cells. We explored the clinical implication of E33 expression in breast patients, providing a novel perspective on preoperative predication of breast cancer. E33 expression was an independent factor of OS in breast cancer patients, and highly expressed LncRNA in diabetic patients without breast cancer may become a crucial factor in the development of breast cancer.

As we all know, breast cancer is a heterogeneous disease at the clinical characteristics and molecular level.^12^, ^13^ Long non‐coding RNA is only one of factors in cancer progression. Even long non‐coding RNA by itself is highly expressed in macrophage in blood. So, whether there is effect of long non‐coding RNA from macrophage would promote cell growth of breast cancer cell is not elucidated. But, long non‐coding RNA is very important for breast cancer patients with type 2 diabetes.

Body Mass Index is an independent factor for diabetes.^3^ And for Asians in United States, the risk of type 2 diabetes is very high when BMI is over 25, but for Whites, the same risk need BMI over 27. So, type 2 diabetes is extremely important factor for women with breast cancer in East Asia including Japan, Korea, Vietnam, and especially China.

E33 has been shown to upregulated in diabetes, and diabetes was a factor of induction of breast cancer. In this study, E33 expression was significantly correlated with TNM stage, lymph node metastasis, and family history, but not correlated with patient's age and tumor grade, as well as ER, PR, and Her2 status.

In conclusion, our data suggest that E33 could be serves as a biomarker and would be a crosslink in breast cancer patients and type 2 diabetes. And it needs to be researched more.
